# Analysis of Aflatoxin Biomarkers in the Hair of Experimental Animals

**DOI:** 10.3390/toxins13080570

**Published:** 2021-08-16

**Authors:** Innocent Mupunga, Ilse Janse van Rensburg, Nokuthula Luthuli, Ovokeroye A. Abafe, Leshweni J. Shai, David R. Katerere

**Affiliations:** 1 Department of Biomedical Sciences, Tshwane University of Technology, Pretoria 0001, South Africa; mupunga@gmail.com (I.M.); ShaiLJ@tut.ac.za (L.J.S.); 2 Faculty of Veterinary Science, University of Pretoria, Pretoria 0028, South Africa; Ilse.JansevanRensburg@up.ac.za; 3 Residue Laboratory, Agricultural Research Centre, Ondersterpoort Veterinary Institute, Pretoria 0110, South Africa; LuthuliN@arc.agric.za (N.L.); AbafeO@arc.agric.za (O.A.A.); 4 Chair in Pharmaceutical and Biotechnological Advancement in Africa, Department of Pharmaceutical Sciences, Tshwane University of Technology, Pretoria 0001, South Africa

**Keywords:** aflatoxin biomarkers, AFM1, AFB1, hair, UHPLC-MS/MS

## Abstract

Analysis of body fluids and tissues of aflatoxin exposed individuals for the presence of aflatoxins and aflatoxin metabolites has emerged as a reliable indicator of exposure and metabolism of aflatoxins. However, current aflatoxin biomarkers are not appropriate for investigating the long-term effects of aflatoxin exposure. In this explorative study, we investigated the analysis of hair as a complementary or alternative matrix for the assessment of biomarkers of long-term aflatoxin exposure. Three groups of guinea pigs were orally dosed with 5 ugkg^−1^bw^−1^, 50 ugkg^−1^bw^−1^, and 100 ugkg^−1^bw^−1^ of AFB1. Urine and hair samples were collected on days 0, 1, 2, 3, 7, 30, 60, and 90 and analysed for AFB1 and AFM1 using UHPLC-MS/MS. AFB1 and AFM1 were detected in 75% and 13.6%, respectively, of the day 1 to day 7 urine samples. AFB1 was detected in hair samples collected from day 3 up to day 60. This is the first report to confirm the deposition of AFB1 in the hair of experimental animals. These findings indicate that hair analysis has the potential to provide an accurate long-term historical record of aflatoxin exposure with potentially important implications for the field of aflatoxin biomarkers.

## 1. Introduction

Aflatoxins are highly toxic and carcinogenic fungal metabolites produced mainly by the *Aspergillus* species that thrive in the hot and humid tropical areas of the world. Acute aflatoxicosis results from consumption of food contaminated with high levels of aflatoxins and has been reported in various African countries for example, in Kenya in 2004 and in Tanzania in 2016 [1,2]. In Tanzania, 68 cases were recorded with a fatality rate of 29.4%, while 317 cases and a fatality rate of 39.4% were reported in Kenya. Media reports indicate that another outbreak might have been experienced in Tanzania in 2019 (http://outbreaknewstoday.com; accessed on 17 June 2021). However, most populations in the developing world are chronically exposed to low levels of aflatoxins that lead to long-term health issues including childhood stunting, reduced immunomodulation, and eventually hepatocellular carcinoma (HCC) [3,4].

Studies of aflatoxin exposure in at risk populations have traditionally been achieved through dietary recall questionnaires and analysis of representative food samples for aflatoxin contamination [5]. However, several limitations make it nearly impossible to translate food analysis and questionnaire results into individual exposure [6,7]. These limitations include a lack of laboratory infrastructure in many settings where high aflatoxin levels are encountered, seasonal variation in aflatoxin contamination of staple foods, and difficulty in estimating individual exposure in settings where families eat from the same plate or bowl of food [7]. Additionally, aflatoxin exposure in the food manufacturing or waste management industries might be due to inhalation or dermal absorption of aflatoxin contaminated dust, and this cannot be monitored through the use of traditional methods [8,9]. Measuring the presence of aflatoxins and aflatoxin metabolites in body fluids and tissues of exposed individuals has emerged as a reliable indicator of exposure and metabolism of aflatoxins [6]. Biomarkers play an important role in hazard characterisation and exposure assessment in cases where other toxins and components in the food matrix affect the bioavailability and system concentrations of aflatoxins [7,10]. Ideal biomarkers are non-invasive, non-destructive, and easily and cheaply measured [7,11].

The use of aflatoxin biomarkers instead of environmental monitoring to assess aflatoxin exposure has several advantages. These include the improved sensitivity and specificity of the measurement of risk factors owing to a reduction in the possibility of misclassification of exposure as the level of exposure is measured rather than relying on the history of exposure [12]. Second, biomarkers measured in biological samples usually reflect integrated effects of repeated exposure, leading to a better understanding of the natural history of disease encompassing the phases of induction, latency, and detection [11,12]. Finally, reliability and validity can be established through pilot studies and statistical analysis.

After ingestion, aflatoxin B1 (AFB1) is metabolised to produce the highly toxic AFB1-8,9-exo-epoxide and less toxic aflatoxicol, aflatoxin M1 (AFM1), aflatoxin P1 (AFP1), and aflatoxin Q1 (AFQ1) [13]. The exo-epoxide may also bind to DNA and protein macromolecules to form AFB1-N7-guanine and AFB1-lysine adducts. AFM1 and AFB1-N7-guanine are known to show a dose-dependent relationship with aflatoxin intake and are also useful markers for assessing individual aflatoxin exposure [5,14]. AFB1-N7-guanine, AFM1, AFP1, AFQ1, and unmetabolized AFB1 are excreted in urine; however, the excretion is rapid and their detection in urine will only reflect 24–48 h post-exposure levels. AFB1-lysine adduct is another metabolite known to show a dose-dependent relationship with aflatoxin intake. It correlates with aflatoxin exposure of 2–3 months based on the half-life of lysine [13,14]. However, it suffers from similar limitations as AFM1 and AFB1-N7-guanine as it only reflects exposure in the last 2–3 months [13]. Additionally, blood collection is an invasive and painful procedure. There are cultural reasons that might discourage people from participating in such invasive studies.

The world of diagnostics is rapidly changing owing to the development of non-invasive tests to diagnose and monitor diseases as well as the increased use of non-invasive matrices in place of blood. Furthermore, the current aflatoxin biomarkers are not stable over the long periods that are required for prospective studies used to investigate the long-term effects of aflatoxin exposure. Non-invasive, easily obtainable matrices including hair and nails have found considerable use in the forensic and drugs of abuse fields as complementary to analysis of traditional matrices and can possibly be adopted as matrices for the analysis of long-term aflatoxin exposure biomarkers [7].

In this paper, we report an explorative study using guinea pigs fed AFB1 to understand if biomarkers are deposited in hair in quantifiable amounts over a period of time.

## 2. Results

### 2.1. Animal Experiments

Dunkin Hartley guinea pigs were randomized into three groups A, B, and C and dosed with 5, 50, and 100 ugkg^−1^bw^−1^ of AFB1, respectively, for 7 consecutive days. They were then monitored daily up until day 90 when the study was terminated. Clinical signs, environmental temperature, humidity, and weekly weights were monitored. Both the control and test animals did not show any adverse clinical signs or any signs of AFB1 toxicity during the study period. The environmental temperature (19–22 °C) and humidity (41–67%) fluctuations observed were within the recommended ranges of 18–24 °C and 40–70% for guinea pigs’ housing temperature and humidity, respectively [15]. Additionally, the average percentage weight gain for all the animals ranged from 90 to 117%. However, there was no significant difference (*p* > 0.05) in average weight gain for control animals and test animals in all three groups and between the test animals in different groups.

Two guinea pigs (one low dose and one high dose animal) were sacrificed on day 8 and sent for post-mortem and histopathological analysis to check for evidence of aflatoxicosis. The low dose animal did not show any signs of toxicity during post-mortem analysis, while the high dose animal showed mild haemorrhage and moderate hepatic and pulmonary congestion. Microscopic examination of both animals’ liver tissues revealed mild granular appearance and mild swelling of the hepatocytes, which could be due to stored toxicological metabolic products [16].

### 2.2. Validation of the Aflatoxins Method

Urine samples were diluted with a mixture of acetonitrile/water and formic acid (0.94/0.05/0.01, *v/v/v*) and directly injected into the UHPLC-MS/MS system. The sample preparation and instrumental methods were optimised to simultaneously detect and quantify both AFB1 and AFM1 in a single run. The method was validated for specificity, linearity, accuracy, and precision. There were no interfering substances at the retention times of interest in both the matrix blanks and spiked samples, indicating good method specificity. The recovery for spiked samples ranged from 82 to 120% for AFB1 and 74 to 131 % for AFM1. The method showed good precision as the relative standard deviation (RSD) for both AFB1 and AFM1 was below 20%. The method had excellent linearity as demonstrated by the co-efficient of regression (R^2^), which was 0.9849 for AFB1 and 0.9764 for AFM1. A summary of the urinary aflatoxins’ method validation results is shown in Table 1.

**Figure 1:**
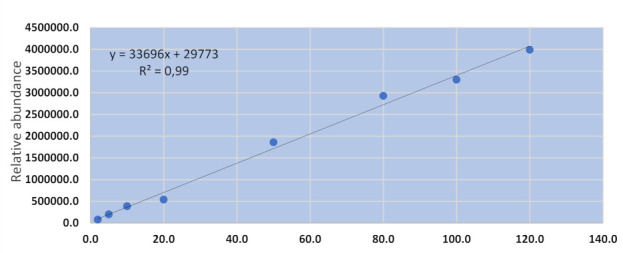
Calibration curve for the AFB1 in hair method.

The AFB1 method for hair analysis was validated by spiking blank hair samples with native AFB1 standard solution (2–120 µgkg^−1^). The method validation results are shown in Table 2 and Figure 1 below.

The LC-MS/MS method was capable of detecting and quantifying AFB1 from spiked samples. The method showed good specificity as the retention times for both the working standard and spiked samples were reproducible (retention time: 3.56 ± 0.01 min). The recovery for spiked samples ranged from 73 to 109%, which showed that the method was accurate for AFB1. The method also showed good precision with an RSD for AFB1 of less than 20%. The method had excellent linearity as demonstrated by the co-efficient of regression (R^2^), which was 0.9969. The linearity of this method was comparable to the results obtained for the LC-MS/MS methods used for the analysis of AFB1 in food commodities (R^2^ > 0.99) and fumonisins in experimental animals and human hair (R^2^ > 0.99) [17,18,19].

### 2.3. Urine Aflatoxins’ Analysis Results

The main objective behind the analysis of urine aflatoxins was to confirm the ingestion, absorption, and biotransformation of AFB1 by the guinea pigs. AFB1 was detected in 66% (33/50) of samples with concentrations ranging from 0.01 to 85.26 µgkg^−1^ with a mean of 20.56 µgkg^−1^, while AFM1 was detected in 12% (6/50) of the day 1 to day 7 urine samples ranging from 0.01 to 3.68 µgkg^−1^ with a mean of 0.96 µgkg^−1^, confirming ingestion and biotransformation of AFB1. None of the control animal urine samples and test animal urine samples from day 0, 30, 60, and 90 had any detectable levels of AFB1 or AFM1, and this confirms that the animals had no prior exposure to AFB1 and that the commercial diet fed to the guinea pigs was AFB1-free.

### 2.4. Hair Aflatoxins’ Analysis Results

**Figure 2:**
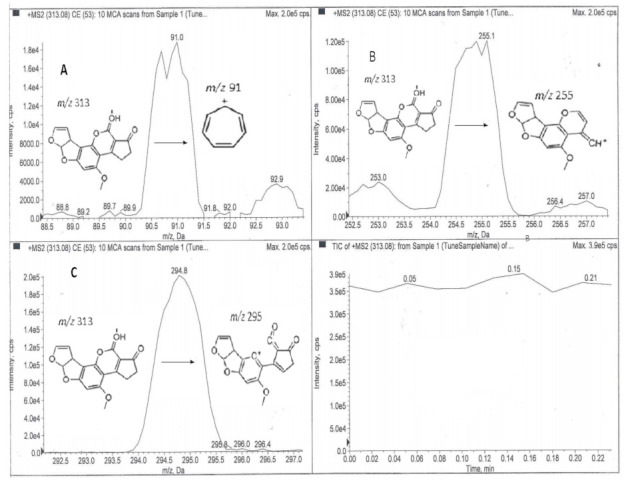
Chromatograms showing AFB1 fragmentation pathways after direct MS/MS analysis of the hair samples: (**A**) 313→91; (**B**) 313→255; (**C**) 313→295.

The hair analysis for the presence of AFB1 was conducted in two parts. The samples were initially analysed via direct MS/MS infusion to qualitatively screen for AFB1, and this was followed by the LC-MS/MS analysis when it became apparent that AFB1 was present in the hair samples. The AFB1 precursor ion was *m/z* 313 and its MS/MS fragmentation led to two known product ions of AFB1 (*m/z* 295 and 255) and a tropylium ion (Figure 2). The fragmentation of 313→295 was most probably due to the loss of H_2_O and the fragmentation 313→255 was probably due to the loss of C_3_H_5_OH. These findings confirm that the parent toxin was detectable in the hair of experimental guinea pigs exposed to AFB1.

These results were also confirmed by the qualitatively detectable levels of AFB1 observed during the LC-MS/MS analysis of the hair samples collected from day 3 up to day 60 (Table 3).

## 3. Discussion

There was no difference in weight gain between the test and control animals, suggesting that the AFB1 dosage used in this study did not affect the animals’ food conversion efficiency, meaning similar weight gain between control animals and test animals. Other researchers, however, reported significant weight differences between control and test animals [20,21]. A dose-dependent decrease in body weight was observed in rats receiving either a single dose of 250 ugkg^−1^ or 1000 ugkg^−1^ or multiple doses of 75 ugkg^−1^ for 5 weeks [20]. A decrease in weight gain from about day 14 in pigs receiving 1.1 mgkg^−1^ body weight of AFB1 and lower weights in test animals compared with control animals in a rabbit study were also reported [21]. However, it must be noted that the current study used AFB1 dosages that were much lower with a shorter duration.

The post-mortem changes observed in the animals sacrificed also confirm that the AFB1 dosage used in the current study was below the levels observed to cause acute aflatoxicosis. The microscopic examination of the liver tissues indicates that toxicological damage took place. The swelling observed in the hepatocytes could be due to stored AFB1 and its metabolites because the liver is the primary site for AFB1 metabolism.

The purpose of the analysis of AFB1 and AFM1 in urine samples was to confirm the ingestion and metabolism of AFB1 by the guinea pigs. The detection of AFB1 in some of the urine samples indicates that AFB1 was absorbed because absorbed AFB1 and its metabolites are excreted in urine, while unabsorbed AFB1 and biliary excretion products are excreted through the faecal route [22]. The detection of urinary AFM1 is also regarded as a reflection of AFB1 ingestion and subsequent metabolism [23]. The undetectable levels from day 0 samples for all the animals confirm that these animals were not exposed to AFB1 through dietary or other means before the commencement of the experimental phase. Furthermore, the undetectable results from day 30, 60, and 90 samples confirmed that urinary AFB1 and AFM1 are short-term biomarkers of aflatoxin exposure and their excretion reflects exposure in the last 24–48 h, as previously reported in the literature [24]. The current results confirm that the guinea pigs ingested and metabolized the AFB1 and excreted AFB1 and AFM1 through the urinary route, and some of the AFB1 was also deposited into the animals’ hair.

To the best of our knowledge, the results of this study describe for the first time the accumulation and detection of AFB1 in the hair of experimental animals, and it is safe to assume that other AFB1 metabolites may also be present in hair in tandem with studies that reported the presence of fumonisins and their metabolites in the hair of humans and experimental animals [17,18,25]. However, in experimental rats, fumonisins were only detected in hair after four weeks [17]. The rats had been divided into four groups; group 1 received a single dose of 1 mgkg^−1^bw^−1^ fumonisin B1, group 2 received a single dose of 10 mgkg^−1^bw^−1^ of fumonisin B1, group 3 received 4.25 mgkg^−1^day^−1^ for 28 days, and group 4 acted as the control group. Fumonisin B1 was detected in the fourth week for group 2 and group 3 animals, while fumonisin B1 was not detected in the hair samples from group 1 animals, implying the dose might have been too low to allow the accumulation of fumonisin B1 in hair samples. In the current study, AFB1 was detected as early as day 3 from all the dosage levels, implying that the bioaccumulation of AFB1 in hair samples might be rapid without a lag period due to its lipophilicity [26]. Similarly, codeine (a drug of abuse), was detected in human beard specimens within 24 h of administration [27].

The mechanisms by which AFB1 and its metabolites are deposited into the hair are not clear; however, there are several possibilities. First, it is possible that they are transported through the blood stream into the hair follicle where they perfuse the hair root and are embedded in the keratinized matrix, as suggested by Sewram et al. for fumonisins [17]. There is a rich blood supply around the hair bulb and its glands, which allow nutrients, waste products, and potential toxins to diffuse into the hair [27]. Second, AFB1 and its metabolites could also be stored in the guinea pigs’ tissues. The toxins are then released slowly into the blood stream before being transported into the hair follicle, which is surrounded by a rich blood supply. The toxins can also be released through transdermal diffusion from the skin and contaminating hair shafts and hair fibres [27]. Hair shafts are embedded about 3 mm below the surface of the epidermal epithelium of the skin and can easily be contaminated by toxins diffusing from the blood stream, tissues, and fat stores through the skin. Third, it is possible that due to their lipophilicity aflatoxins are stored in the guinea pigs’ adipose tissues. They are then released as sweat, sebum, and other bodily secretions that can bath the hair follicle, leading to auto contamination of the guinea pigs’ hair [17,27]. The apocrine and eccrine sweat glands and the sebaceous glands secrete fluids that coat the skin and keratinized matrices [27]. The eccrine sweat glands are very close to the hair follicle, while the sebaceous and apocrine glands have ducts that open directly into the hair follicle. The sweat and sebum introduce substances such as water, lipids, inorganic salts, waste products, and potentially aflatoxins into the hair. This mechanism, however, would have been highly unlikely in this study because guinea pigs do not have sweat glands and cannot regulate heat through sweating [15]. Additionally, the tightly controlled temperature conditions during the study would have meant the guinea pigs were always in a comfortable environment. Finally, there is a possibility that the AFB1 detected in the hair samples could have been due to environmental contamination. Unreacted AFB1, AFM1, AFB1-N7-guanine, AFP1, and AFQ1 are all excreted in the urine of exposed animals, and it is probable that the urine and faeces may contaminate the bedding and in turn contaminate the animals’ hair. However, this possibility is unlikely as the bedding was changed weekly and the hair samples were washed soon after collection as well as just before extraction and clean-up.

Based on these results, there is a possibility that AFB1 can also be detected in human hair. Fumonisin mycotoxins have been detected in human hair samples from Brazil and South Africa and the probable daily intakes of fumonisin B1 could be calculated from the fumonisin B1 levels in hair samples [18,25]. The detection of aflatoxins in experimental animals coupled with detection of fumonisins in experimental animals and human hair shows that mycotoxins can accumulate in hair samples. Further studies will be required before AFB1 in hair can be used as a matrix for biomarkers of both short- and long-term aflatoxin exposure and epidemiological studies in humans. These may include studies to confirm the dose–response relationship between AFB1 intake and AFB1 levels in hair samples, the relationship between AFB1 in hair and disease onset, as well as confirmation of the duration of AFB1 retention in hair samples.

Even though the results of this study are ground-breaking, a number of limitations are acknowledged, and the results should be interpreted in light of these shortcomings. To begin with, the lack of authentic commercial AFB1-lysine and AFB1-N7-guanine standards meant only AFB1 and AFM1 in urine were analysed. AFB1 and AFM1 were detected in the urine samples, confirming the ingestion, absorption, metabolism, and excretion of AFB1 by the guinea pigs.

## 4. Conclusions

As far as we know, this is the first report to confirm the deposition of AFB1 in the hair of experimental animals. It appears that aflatoxins and their metabolites can be incorporated into the hair, which then grows continuously at a fairly constant rate; hence, hair analysis has the potential to provide an accurate long-term historical record of aflatoxin exposure. This has potentially important implications for the field of aflatoxin biomarkers. Hair analysis can be used as a complimentary matrix to the traditional blood and urine analysis or as a stand-alone method where aflatoxin exposure might have occurred weeks or months prior to sample collection and in cases where retrospective epidemiological investigations are being carried out. Hair analysis allows for retrospective investigation of aflatoxin exposure long after the aflatoxins have been eliminated from the body. The advantages of using hair samples include the following: (i) sample collection is easy and non-invasive; (ii) sample collection can be performed under close supervision; and (iii) samples can be transported and stored at room temperature, making it an ideal sample type in most remote areas. However, further studies will be required to confirm these ground-breaking findings as well as to improve our understanding of the significance of AFB1 and its metabolites in the hair of humans and animals.

## 5. Materials and Methods

### 5.1. Equipment, Chemicals and Reagents

All the chemicals used in this work were of high-performance liquid chromatography (HPLC) grade and were purchased from Merck Chemicals (Darmstadt, Germany) unless otherwise stated. The AFB1 and AFM1 standards were purchased from The Council for Scientific and Industrial Research (CSIR), Pretoria, South Africa. Chromatographic separation was carried out with Shimadzu Nexera X2 LC-30AD with autosampler CTO30A, SIL−30AC, CBM–20A (Shimadzu, Kyoto, Japan), while MS/MS was carried out with an ABSCIEX 4500 series quadrupole ion trap mass spectrometer (Absciex LLC, Framingham, MA, USA) operated with Analyst^®^ Software version (1.5.1) and MultiQuant^®^ for quantitation. The mass spectrometer was fitted with an electrospray ionization (ESI) turbo spray source operating in the positive polarity mode. The ion spray voltage was set at −5500 V and an entrance potential of −10 V. The multiple reaction monitoring (MRM) mode was utilized for data acquisition. The settling time and mass range pause were 0 ms and 5 ms, respectively. The chromatographic separation of aflatoxins was achieved on a Kinetex XB-C18 column (50 mm × 2.1 mm, 2.6 µm) (Phenomenex, Torrance, CA, USA). The mobile phase consisted of A (5 mM Ammonium fluoride/water *v/v*) and B (5 mM ammonium fluoride/methanol *v/v*). The gradient condition started with 100 % A for 4 min, followed by 100 % B for a further 4 min, and then equilibrated for 3 min with the initial mobile phase condition of 100 % A before each chromatographic run. The flow rate was 500 µLmin^−1^.

### 5.2. Animal Experiment

All experimental procedures involving animals were consistent with the South African law and were approved by the University of South Africa College of Agriculture and Environmental Sciences Animal Ethics Review Committee on 12 June 2014 (permit number: 2014/CAES/052).

White Dunkin Hartley guinea pigs were used in this study. Eighteen animals were divided into three groups (group 1—low dose, group 2—medium dose, and group 3—high dose) of six animals each. All the animals underwent clinical examination by a qualified veterinary surgeon before commencement of the study and were certified as healthy to be included in the study. The animals were housed under controlled conditions of temperature, humidity, and lighting. They were housed in groups inside solid floor cages and were only placed in individual metabolic cages during sample collection and returned to the group immediately after sample collection. Room temperature and relative humidity in the animal housing were monitored daily. Food and water were available ad libitum.

A known amount of the AFB1 stock solution was dissolved and mixed in corn oil and orally administered via gavage once a day for 7 consecutive days to the respective treatment groups. The AFB1 dosages were 5 µgkg^−1^, 50 µgkg^−1^, and 100 µgkg^−1^ of body weight for group 1, group 2, and group 3 animals, respectively. Two animals in each group acted as the control and received only corn oil. After the dosage phase was completed, the animals then received a certified AFB1 free diet up to day 90 and they were weighed once a week for the duration of the study. Additionally, the animals received fresh fruits and vegetables daily.

Hair and urine samples were collected from randomly selected animals just before treatment (this became day 0) and then from all the animals on the following days: day 3, 7, 30, 60, and 90. Urine samples were also collected on day 1 and day 2. For urine collection, the animals were placed into metabolic cages (Techniplast, Milan, Italy) over a 24 h period and the urine was collected into flasks immersed in dry acetone, with the collection day marked. Hair samples were collected from pre-defined areas using an electric shaver and then cut into small pieces, washed in distilled water, and dried at 40 °C. Two animals (one high dose and one low dose animal) were sacrificed on day 8 and sent for postmortem and histopathological analysis by a commercial veterinary pathology laboratory.

### 5.3. Sample Analysis

The sample preparation methodology for AFB1 and AFM1 in urine followed a previously described “dilute and shoot” procedure with some modifications [28]. In brief, urine samples were thawed and allowed to reach room temperature. A 600 µL aliquot was centrifuged in a 5415R Eppendorf refrigerated centrifuge (Epperndorf, Hamburg, Germany) at 13,000 rpm for 10 min at 4 °C. One hundred microliters (100 uL) of the supernatant was diluted with 900 µL of water/acetonitrile/formic acid (0.94/0.05/0.01, *v/v/v*). The diluted mixture was directly injected onto the UHPLC-MS/MS for the analysis of AFB1 and AFM1. Other urinary metabolites were not assessed as authentic commercial standards for these were not available.

Hair samples were analyzed by direct MS/MS infusion and UHPLC-MS/MS for AFB1 after extraction and clean-up. Extraction and clean-up for the analysis of AFB1 in hair samples was done using a previously validated in-house method for the extraction of food and feed. In brief, hair samples were mixed with sodium chloride (5:1 *w/w*) and extracted with 70% methanol by shaking for 30 min on a Platform Shaker (Labotec, Midrand, South Africa). The extracts were then centrifuged (Allegra X-22R, Beckman Coulter, Chaska, MN, USA) at 5600 rpm for 10 min at 4 °C. The supernatant was filtered with Whatman No. 4 filter paper (Whatman, Maidstone, UK) and 10 mL of the filtrate was diluted with 20 mL of phosphate buffer (pH 7.4). The mixture was applied onto AflaTest^®^ Immuno-affinity Columns (mounted on an SPE manifold connected to a vacuum pump) at a rate of 1–2 drops per second. The columns were rinsed with 10 mL of distilled water and dried under vacuum. AFB1 was eluted under vacuum using two aliquots of 1 mL methanol into amber vials and 1 mL of HPLC grade water was added to the eluate. The diluted eluate was filtered through 0.45 µm syringe filters before MS/MS direct infusion and UHPLC-MS/MS analysis. Direct MS/MS infusion was carried with an electrospray ionization mass spectrometer in the positive polarity mode. The search range for the precursor ion was set between 150.00 and 550.00 Daltons. The known AFB1 base peak was selected and optimized. The product ions were selected from the three most intense peaks. Product ions within ±10 Daltons of the precursor ion mass-to-charge ratio (*m/z*) were excluded.

### 5.4. Method Validation

UHPLC-MS/MS methods for urinary and hair aflatoxins were assessed for specificity, linearity, accuracy, and precision. The specificity of the method was determined by verifying the retention times of each compound in both matrix blank and spiked blank samples (n = 3). The absence of interfering compounds verifies the specificity of the method. The linearity of the method was determined with an eight-point matrix-matched calibration curve over a concentration range of 20–120 µgkg^−1^. The accuracy and precision were determined by the analysis of spiked samples (n = 3) at three different concentrations indicating low, average, and high spiked levels. Samples that previously tested negative for the target compounds were used as matrix blanks utilized for the preparation of calibration curve and validation of the analytical method.

### 5.5. Data Analysis

The data obtained from different experiments were analyzed using Excel 2013 (Microsoft Corporation, Washington, DC, USA) and presented as tables, graphs, and charts.

## Figures and Tables

**Table 1:** Summary of the validation results of the urine aflatoxins’ LC-MS/MS method. RSD, relative standard deviation.

Analyte	Validation Range (µgkg^−1^)	Recovery (%)	R^2^ Value	Retention Time (mins)	RSD	Limit of Quantitation (µgkg^−1^)
AFB1	2–120	82–120	0.9849	3.58 (±0.01)	12.37	0.01
AFM1	2–120	74–131	0.9764	3.43 (±0.01)	19.23	0.01

**Table 2:** Summary of the hair method validation results.

Analyte	Validation Range (µgkg^−1^)	Recovery (%)	R^2^ Value	Retention Time (mins)	RSD	Limit of Quantitation (µgkg^−1^)
AFB1	2–120	73–108	0.9969	3.55 (±0.07)	12.37	0.01

**Table 3:** The LC-MS/MS AFB1 results in hair.

Pooled sample	AFB1 results
Day 3—Low dose	Detected
Day 3—Medium dose	Detected
Day 3—High dose	Detected
As PDDay 7—Low dose	Detected
Day 7—Medium dose	Detected
Day 7—High dose	Detected
Day 30—Low dose	Not detected
Day 30—Medium dose	Detected
Day 30—High dose	Detected
Day 60—Low dose	Not detected
Day 60—Medium dose	Not detected
Day 60—High dose	Detected
Day 90—Low dose	Not detected
Day 90—Medium dose	Not detected
Day 90—High dose	Not detected

## Data Availability

Not applicable.
